# Designs for clinical trials with time-to-event outcomes based on stopping guidelines for lack of benefit

**DOI:** 10.1186/1745-6215-12-81

**Published:** 2011-03-18

**Authors:** Patrick Royston, Friederike M-S Barthel, Mahesh KB Parmar, Babak Choodari-Oskooei, Valerie Isham

**Affiliations:** 1 MRC Clinical Trials Unit 222 Euston Road London NW1 2DA UK; 2 Department of Statistical Science University College London 1-19 Torrington Place London WC1E 6BT UK

## Abstract

**background:**

The pace of novel medical treatments and approaches to therapy has accelerated in recent years. Unfortunately, many potential therapeutic advances do not fulfil their promise when subjected to randomized controlled trials. It is therefore highly desirable to speed up the process of evaluating new treatment options, particularly in phase II and phase III trials. To help realize such an aim, in 2003, Royston and colleagues proposed a class of multi-arm, two-stage trial designs intended to eliminate poorly performing contenders at a first stage (point in time). Only treatments showing a predefined degree of advantage against a control treatment were allowed through to a second stage. Arms that survived the first-stage comparison on an intermediate outcome measure entered a second stage of patient accrual, culminating in comparisons against control on the definitive outcome measure. The intermediate outcome is typically on the causal pathway to the definitive outcome (i.e. the features that cause an intermediate event also tend to cause a definitive event), an example in cancer being progression-free and overall survival. Although the 2003 paper alluded to multi-arm trials, most of the essential design features concerned only two-arm trials. Here, we extend the two-arm designs to allow an arbitrary number of stages, thereby increasing flexibility by building in several 'looks' at the accumulating data. Such trials can terminate at any of the intermediate stages or the final stage.

**Methods:**

We describe the trial design and the mathematics required to obtain the timing of the 'looks' and the overall significance level and power of the design. We support our results by extensive simulation studies. As an example, we discuss the design of the STAMPEDE trial in prostate cancer.

**Results:**

The mathematical results on significance level and power are confirmed by the computer simulations. Our approach compares favourably with methodology based on beta spending functions and on monitoring only a primary outcome measure for lack of benefit of the new treatment.

**Conclusions:**

The new designs are practical and are supported by theory. They hold considerable promise for speeding up the evaluation of new treatments in phase II and III trials.

## 1 Introduction

The ongoing developments in molecular sciences have increased our understanding of many serious diseases, including cancer, HIV and heart disease, resulting in many potential new therapies. However, the US Food and Drug Administration has identified a slowdown, rather than an expected acceleration, in innovative medical therapies actually reaching patients [1]. There are probably two primary reasons for this. First, most new treatments show no clear advantage, or at best have a modest effect, when compared with the current standard of care. Second, the large number of such potential therapies requires a corresponding number of large and often lengthy clinical trials. The FDA called for a 'product-development toolkit' to speed up the evaluation of potential treatments, including novel clinical trial designs. As many therapies are shown not to be effective, one component of the toolkit is methods in which a trial is stopped 'early' for lack of benefit or futility.

Several methodologies have been proposed in the past to deal with stopping for futility or lack of benefit, including conditional power and spending functions. With the futility approach, assumptions are made about the distribution of trial data yet to be seen, given the data so far. At certain points during the trial, the conditional power is computed, the aim being to quantify the chance of a statistically significant final result given the data available so far. The procedure is also known as stochastic curtailment. As a sensitivity analysis, the calculations may be carried out under different assumptions about the data that could be seen if the trial were continued [2]. For example, treatment effects of different magnitudes might be investigated under the alternative hypothesis of a non-null treatment effect.

Alpha-spending functions were initially proposed by Armitage et al. [3] and extensions to the shape of these functions were suggested by several authors including Lan & DeMets [4] and O'Brien & Fleming [5]. In essence, the approach suggests a functional form for 'spending' the type 1 error rate at several interim analyses such that the overall type 1 error is preserved, usually at 5%. The aim is to assess whether there is evidence that the experimental treatment is superior to control at one of the interim analyses. Pampallona et al. [6] extended the idea to beta or type 2 error spending functions, potentially allowing the trial to be stopped early for lack of benefit of the experimental treatment.

In the context of stopping for lack of benefit, Royston et al. [7] proposed a design for studies with a time-to-event outcome that employs an intermediate outcome in the first stage of a two-stage trial with multiple research arms. The main aims are quickly and reliably to reject new therapies unlikely to provide a predefined advantage over control and to identify those more likely to be better than control in terms of a definitive outcome measure. An experimental treatment is eliminated at the first stage if it does not show a predefined degree of advantage (e.g. a sufficiently small hazard ratio) over the control treatment. In the first stage, an experimental arm is compared with the control arm on an intermediate outcome measure, typically using a relaxed significance level and high power. The relaxed significance level allows the first stage to end relatively early in the trial timeline, and high power guards against incorrectly discarding an effective treatment. Arms which survive the comparison enter a further stage of patient accrual, culminating at the end of the second stage in a comparison against control based on the definitive outcome.

A multi-arm, two-stage design was used in GOG182/ICON5 [8], the first such trial ever run. Early termination indeed occurred for all the experimental arms. The trial, which compared four treatments for advanced ovarian cancer against control, was conducted by the Gynecologic Oncology Group in the USA and the MRC Clinical Trials Unit, London, and investigators in Italy and Australia. The trial was planned to run in two stages, but after the first-stage analysis, the Independent Data Monitoring Committee saw no justification to continue accrual to any of the treatment arms based on the intermediate outcome of progression-free survival. Early stopping allowed resources to be concentrated on other trials, hypothetically saving about 20 years of trial time compared with running four two-arm trials one after the other with overall survival as the primary outcome measure.

Here, we show how a parallel group, two-arm, two-stage design may be extended to three or more stages, thus providing stopping guidelines at every stage. Designs with more than two arms involve several pairwise comparisons with control rather than just one; apart from the multiplicity issue, the multi-arm designs are identical to the two-arm designs. In the present paper, section 2 describes the designs and the methodology underlying our approach, including choice of outcome measure and sample size calculation. Section 3 briefly compares our approach with designs based on beta-spending functions. In section 4, we present simulation studies to assess the operating characteristics of the designs in particular situations. In section 5, we describe a real example, the ongoing MRC STAMPEDE [9] randomized trial in prostate cancer, which has six arms and is planned to run in 5 stages. The needs of STAMPEDE prompted extension of the original methodology to more than two stages. Further design issues are discussed in section 6.

## 2 Methods

### 2.1 Choosing an intermediate outcome measure

Appropriate choices of an intermediate outcome measure (*I*) and definitive outcome measure (*D*) are key to the design of our multi-stage trials. Without ambiguity, we use the letters *I *and *D *to mean either an outcome measure (i.e. time to a relevant event) or an outcome (an event itself), for example *I *= (time to) disease progression, *D *= (time to) death. The 'treatment effect' on *I *is *not *required to be a surrogate for the treatment effect on *D*. The basic assumptions for *I *in our design are that it occurs no later than *D*, more frequently than *D *and is on the causal pathway to *D*. If the null hypothesis is true for *I*, it must also hold for *D*.

Crucially, it is not necessary that a true alternative hypothesis for *I *translate into a true alternative hypothesis for *D*. However, the converse must hold - a true alternative hypothesis for *D *must imply a true alternative hypothesis for *I*. Experience tells us that it is common for the magnitude of the treatment effect on *I *to exceed that on *D*.

As an example, consider the case mentioned above, common in cancer, in which *I *= time to progression or death, *D *= time to death. It is quite conceivable for a treatment to slow down or temporarily halt tumour growth, but not ultimately to delay death. It would of course be a problem if the reverse occurred and went unrecognised, since the power to detect the treatment effect on *I *in the early stages of one of our trials would be compromised, leading to a larger probability of stopping the trial for apparent lack of benefit. In practice, we typically make the conservative assumption that the size of the treatment effect is the same on the *I *and *D *outcomes.

In the latter case, a rational choice of *I *might be *D *itself. The case *I *= *D *is also relevant to other practical situations, for example the absence of an obvious choice for *I*, and is a special case of the methodology presented here.

The treatment effects, i.e. (log) hazard ratios, on *I *and *D *do not need to be highly correlated, although in practice they often are. We refer here to the correlation between treatment effects on *I *and *D within the trial*, not across cognate trials. When *I *and *D *are time-to-event outcome measures, the correlation of the (log) hazard ratios is time-dependent. Specifically, the correlation depends on the accumulated numbers of events at different times, as discussed in section 2.7.

Examples of intermediate and primary outcome measures are progression-free (or disease-free) survival and overall survival for many cancer trials, and CD4 count and disease-specific survival for HIV trials.

### 2.2 Design and sample size

Our multi-arm, multi-stage (MAMS) designs involve the pairwise comparison of each of several experimental arms with control. In essence, we view MAMS designs as a combination of two-arm, multi-stage (TAMS) trials; that is, we are primarily interested in comparing each of the experimental arms with the control arm. Apart from the obvious issue of multiple treatment comparisons, methodological aspects are similar in MAMS and TAMS trials. In this paper, therefore, we restrict attention to TAMS trials with just one experimental arm, *E*, and a control arm, *C*.

Assume that the definitive outcome measure, *D*, in a randomized controlled trial is a time- and disease-related event. In many trials, *D *would be death. As just discussed, in our multi-stage trial design we also require a time-related intermediate outcome, *I*, which is assumed to precede *D*.

A TAMS design has *s *> 1 stages. The first *s *- 1 stages include a comparison between *E *and *C *on the intermediate outcome, *I*, and the *s*th stage a comparison between *E *and *C *on the definitive outcome, *D*. Let Δ*_i _*be the true hazard ratio for comparing *E *with *C *on *I *at the *i*th stage (*i *<*s*), and let Δ*_s _*be the true hazard ratio for comparing *E *with *C *on *D *at the *s*th stage. We assume proportional hazards holds for all treatment comparisons.

The null and alternative hypotheses for a TAMS design are

The primary null and alternative hypotheses, *H*_0 _(stage *s*) and *H*_1 _(stage *s*), concern Δ*_s_*, with the hypotheses at stage *i *(*i *<*s*) playing a subsidiary role. Nevertheless, it is necessary to supply design values for all the hypotheses. In practice, the  are almost always taken as 1 and the  as some fixed value < 1 for all *i *= 1, ..., *s*; in cancer trials,  = 0.75 is a often reasonable choice. Note, however, that taking  for all *i *<*s *is a conservative choice; the design allows for . For example, in cancer, if *I *is progression-free survival and *D *is death it may be realistic and efficient to take, say,  = 0.75 and  = 0.7 for *i *<*s*. In what follows, when the interpretation is clear we omit the (stage *i*) qualifier and refer simply to *H*_0 _and *H*_1_.

If *E *is better than *C *then  for all *i*. Let  be the estimated hazard ratio comparing *E *with *C *on outcome *I *for all patients recruited up to and including stage *i*, and  be the estimated hazard ratio comparing *E *with *C *on *D *for all patients at stage *s *(i.e. at the time of the analysis of the definitive outcome).

The allocation ratio, i.e. the number of patients allocated to *E *for every patient allocated to *C*, is assumed to be *A*, with *A *= 1 representing equal allocation, *A *< 1 relatively fewer patients allocated to *E *and *A *> 1 relatively more patients allocated to *E*.

The trial design with a maximum of *s *stages screens *E *for 'lack of benefit' at each stage, as follows:

**Stages 1 to ***s *- 1

1. For stage *i*, specify a significance level *α_i _*and power *ω_i _*together with hazard ratios  and , as described above.

2. Using the above four values, we can calculate *e_i_*, the cumulative number of events to be observed in the control arm during stages 1 through *i*. Consequently, given the accrual rate, *r_i_*, and the hazard rate, *λ_I_*, for the *I*-outcome in the control arm, we can calculate *n_i_*, the number of patients to be entered in the control arm during stage *i*, and *An_i_*, the corresponding number of patients in the experimental arm. We can also calculate the (calendar) time, *t_i_*, of the end of stage *i*.

3. Given the above values, we can also calculate a critical value, *δ_i_*, for rejecting *H*_0 _= Δ*_i _*= . We discuss the determination of *δ_i _*in detail in section 2.3.

4. At stage *i*, we stop the trial for lack of benefit of *E *over *C *if the estimated hazard ratio, , exceeds the critical value, *δ_i_*. Otherwise we continue to the next stage of recruitment.

**Stage ***s*:

The same principles apply to stage *s *as to stages 1 to *s *- 1, with the obvious difference that *e_s_*, the required number of control arm events (cumulative over all stages), and λ*_D_*, the hazard rate, apply to *D *rather than *I*.

If the experimental arm survives all of the *s *- 1 tests at step 4 above, the trial proceeds to the final stage, otherwise recruitment is terminated early.

To limit the total number of patients in the trial, an option is to stop recruitment at a predefined time, t*, during the final stage. Stopping recruitment early increases the length of the final stage. See Appendix A for further details.

To implement such a design in practice, we require values for *δ_i_*, *e_i_*, *n_i _*for stages *i *= 1, ..., *s*. To plan the trial timelines, we also need *t*_1_, ..., *t*_s_, the endpoints of each stage. We now consider how these values are determined.

### 2.3 Determining the critical values *δ*_1_, ..., *δ_s_*

We assume that the estimated log hazard ratio, ln , at stage *i *is distributed as follows:

where  and  are approximate variances under *H*_0 _and *H*_1_, respectively. Suppose that *α*_1_, ..., *α_s_*, one-sided significance levels relevant to these hypotheses, have been specified. By definition

say, where  with superscript 0 or 1 denotes the square root of the relevant  and Φ(·) is the standard normal distribution function. Similarly, specifying powers (one minus type 2 error probabilities) *ω*_1_, ..., *ω_s_*, we have(1)(2)

It follows that

To obtain the critical values, *δ_i_*, it is necessary to provide values of the significance level, *α_i_*, and power, *ω_i_*, for every stage. We discuss the choice of these quantities in section 2.6.

We also need values for  and . According to Tsiatis [10], the variance of ln  under *H*_0 _or under *H*_1 _is given approximately by(3)

where *A *is the allocation ratio, *e_i _*is the number of *I*-events at stage *i *= 1, ..., *s *- 1 and *e_s _*is the number of *D*-events at stage *s *in the control arm (see section 2.2). It follows that(4)

Under *H*_1 _there are fewer events of both types than under *H*_0_, and therefore the power undershoots the desired nominal value, *ω_i_*. A better estimate of the power is based on a more accurate approximation to the variance of a log hazard ratio under *H*_1_, namely, the sum of the reciprocals of the numbers of events in each arm, allowing for the smaller number expected under *H*_1_. We therefore take  as in eqn. (3) and(5)

where  is the number of events in the experimental arm under *H*_1 _by the end of stage *i *when there are *e_i _*events in the control arm and the allocation ratio is *A*. (Note that *A *is implicitly taken into account in .) An algorithm to calculate *e_i_*,  and the corresponding *t_i _*is described next.

### 2.4 Algorithm to determine number of events and duration of stages

The values of *e_i_*,  and *t_i _*for *i *= 1, ..., *s *are found by applying an iterative algorithm, which in outline is as follows:

1. Use eqn. (4) to calculate an initial estimate of *e_i_*, the number of events required in the control arm.

2. Calculate the corresponding critical log hazard ratio .

3. Calculate *t_i_*, the time at which stage *i *ends.

4. Calculate under *H*_1 _the numbers of events expected in the control arm (*e_i_*) and experimental arm () by time *t_i_*.

5. Using eqn. (1), calculate , the power at the end of stage *i *available with *e_i _*and  events.

6. If , increment *e_i _*by 1 and return to step 2, otherwise terminate the algorithm.

Details of two subsidiary algorithms required to implement steps 3 and 4 are given in Appendix A.

Note that the above algorithm requires only the proportional hazards assumption in all calculations except that for the stage end-times, *t_i_*, where we assume that times to *I *and to *D *events are exponentially distributed. The exponential assumption is clearly restrictive, but if it is breached, the effect is only to reduce the accuracy of the *t_i_*. The key design quantities, the numbers (*e_i _*and ) of events required at each stage, are unaffected.

### 2.5 Determining the required numbers of patients

A key parameter of the TAMS design is the anticipated patient recruitment (or accrual) rate. Let *r_i _*be the number of patients entering the control arm per unit time during stage *i*. Accrual is assumed to occur at a uniform rate in a given stage. In practice, *r_i _*tends to increase with *i *as recruitment typically picks up gradually during a trial's life cycle. Let *t*_0 _= 0, and let *d_i _*= *t_i _*- *t_i _*_- 1 _(*i *= 1, ..., *s*) be the duration of the *i*th stage. The number of patients recruited to the control arm during stage *i *is *n_i _*= *r_i_d_i_*, and to the experimental arm it is *An_i_*. Provided that *E *'survives' all *s *- 1 intermediate stages, the total number of patients recruited to the trial is .

To limit the required sample size, the trialist may plan to halt recruitment at a time t* <*t_s _*which occurs during some stage *a *+ 1 (0 ≤ *a *<*s*), and follow the patients up until the required number of events is observed. However, halting recruitment before the end of any intermediate stage would remove the possibility of ceasing recruitment to experimental arms during that or later stages, thus making those stages redundant. The only sensible choice, therefore, is for t* to occur during the final stage, and we can take *a *= *s *- 1. The required number of patients is then

where d* = t* - *t*_*s*-1 _and t* is taken as *t_s _*if recruitment continues to the end of stage *s*.

### 2.6 Setting the significance level and power for each stage

Reaching the end of stage *i *(*i *<*s*) of a TAMS trial triggers an interim analysis of the accumulated trial data, the outcome of which is a decision to continue recruitment or to terminate the trial for lack of benefit. The choice of values for each *α_i _*and *ω_i _*at the design stage is guided by two considerations.

First, we believe it is essential to maintain a high overall power (*ω*) of the trial. The implication is that for testing the treatment effect on the intermediate outcome, the power *ω_i _*(*i *<*s*) should be high, e.g. at least 0.95. For testing the treatment effect on the definitive outcome, the power at the *s*th stage, *ω_s_*, should also be high, perhaps of the order of at least 0.9. The main cost of using a larger number of stages is a reduction in overall power.

Second, given the *ω_i_*, the values chosen for the *α_i _*largely govern the numbers of events required to be seen at each stage and the stage durations. Here we consider larger-than-traditional values of *α_i_*, because we want to make decisions on dropping arms reasonably early, i.e. when a relatively small number of events has accrued. Given the magnitude of the targeted treatment effect and our requirement for high power, we are free to change only the *α_i_*. It is necessary to use descending values of *α*_*i*_, otherwise some of the stages become redundant. For practical purposes, a design might be planned to have roughly equally spaced numbers of events occurring at roughly equally spaced times. For example, total (i.e. control + experimental arm) events at stage *i *might be of the order of 100*i*. A geometric descending sequence of *α_i _*values starting at *α*_1 _= 0.5 very broadly achieves these aims. As a reasonable starting point for trials with up to 6 stages, we suggest considering *α_i _*= 0.5*^i ^*(*i *<*s*) and *α_s _*= 0.025. The latter mimics the conventional 0.05 two-sided significance level for tests on the *D*-outcome. More than 6 stages will rarely be needed as they are unlikely to be of practical value.

As an example, Table 1 shows the numbers of events and stage times for two scenarios. *s *= 4 stages, accrual *r_i _*= 100 patients/yr,  = 1,  = 0.75 for *i *= 1, ..., *s*, median survival time for *I *(*D*) events = 1 (2) yr (i.e. hazard *λ_I _*= 0.69, *λ_D _*= 0.35), *α_i _*= 0.5*^i ^*(*i *= 1, 2, 3), *α*_4 _= 0.025, and allocation ratio *A *= 1 or 0.5. Clearly, 'fine-tuning' may be needed, for example reducing *α*_3 _in order to increase *t*_3_.

**Table 1:** Suggested significance level and power at each stage of a TAMS design with four stages and an allocation ratio of either 1 or 0.5.

Allocation Ratio	Stage	Significance level (1-sided)	Power	Number of events		Time
				Control arm	Total	
*A*	*i*	*α_i_*	*ω_i_*	*e_i_*		*t_i_*
1	1	0.5	0.95	73	133	1.7
	2	0.25	0.95	139	256	2.6
	3	0.125	0.95	198	369	3.3
	4	0.025	0.9	264	486	5.0
						
0.5	1	0.5	0.95	113	160	1.9
	2	0.25	0.95	211	301	2.8
	3	0.125	0.95	301	432	3.6
	4	0.025	0.9	399	568	5.4

The number of events in the control arm and overall at each stage are shown, together with the time at which each stage ends. The assumptions underlying the calculations are described in the text.

### 2.7 Determining the overall significance level and power

Having specified the significance level and power for each stage of a TAMS design, the overall significance level, *α*, and power, *ω*, are required. They are defined as

We assume that the distribution of  is multivariate normal with the same correlation matrix, *R*, under *H*_0 _and *H*_1_. We discuss the meaning and estimation of *R *below. In the notation of section 2.3, we have(6)

where Φ*_s_*(.;*R*) denotes the standard *s*-dimensional multivariate normal distribution function with correlation matrix *R*.

The (*i*, *j*)th element *R_ij _*of *R *(*i*, *j *= 1, ..., *s*) is the correlation between  and , the log hazard ratios of the outcome measures at the ends of stages *i *and *j*. For *i*, *j *<*s *we show in Appendix B that, to an excellent first approximation,(7)

Since  and  are asymptotically equal, our approximation to *R_ij _*is

Exact calculation of the correlation *R_is _*between the log hazard ratios on the *I*- and *D*-outcomes appears intractable. It depends on the interval between *t_i _*and *t_s _*and on how strongly related the treatment effects on the *I *and *D *outcomes are. If *I *is a composite event which includes *D *as a subevent (for example, *I *= progression or death, *D *= death), the correlation could be quite high. In section 2.7.1 we suggest an approach to determining *R_is _*heuristically.

If the *I *and *D *outcomes are identical, *α *and *ω *in eqn. (6) are the overall significance level and power of a TAMS trial. When *I *and *D *differ, the overall significance level, *α_I_*, and power, *ω_I_*, of the combined *I*-stages only are

where *R*^(*s*-1) ^denotes the matrix comprising the first *s *- 1 rows and columns of *R*. Even with no information on the values of *R_is_*, lower and upper bounds on *α *and *ω *may be computed as

The minima occur when *R_is _*= 1 for all *i *(i.e. 100% correlation between  and ), and the maxima when *R_is _*= 0 for all *i *(no correlation between  and ).

Note that unlike for standard trials in which *α *and *ω *play a primary role, neither *α *nor *ω *is required to realize a TAMS design. However, they still provide important design information, as their calculated values may lead one to change the *α_i _*and/or the *ω_i_*.

#### 2.7.1 Determining *R_is_*

In practice, values of *R_is _*are unlikely to lie close to either 0 or 1. One option, as described in Reference [7], is to estimate *R_is _*by bootstrapping relevant existing trial data after the appropriate numbers of *I*-events or *D*-events have been observed at the end of the stages of interest. The approach is impractical as a general solution, for example for implementation in software.

An alternative, heuristic approach to determining *R_is _*is as follows. Given the design parameters (*α_i_*, *ω_i_*) (*i *= 1, ..., *s*), the number *e_i _*of control-arm *I*-events is about the same as the number of *D*-events, when the calculations are run first using only *I*-outcomes and then using only *D*-outcomes. (Essentially, the two designs are the same.) Therefore, the correlation structure of the hazard ratios between stages must be similar for *I*-events and *D*-events. For designs in which *I *and *D *differ, we conjecture that(8)

where *c *is a constant independent of the stage, *i*. We speculate that *c *is related to , the correlation between the estimated log hazard ratios on the two outcomes at a fixed time-point in the evolution of the trial. Under the assumption of proportional hazards of the treatment effect on both outcomes, the expectation of  is independent of time, and can be estimated by bootstrapping suitable trial data [7].

Note that if the *I*- and *D*-outcomes are identical then *c *= 1 and eqn. (8) reduces to eqn. (7). If they are different, the correlation must be smaller and *c *< 1 is an attenuation factor.

We estimated *c *and investigated whether *c *is independent of *i *in a limited simulation study. The design was as described in section 4.3.1. The underlying correlation between the normal distributions used to generate the exponential time-to-event distributions for *I*- and *D*-events was 0.6. The value of *c *was estimated as  for the first two combinations of *α_i _*(the third combination produces a degenerate design when only *I*-events are considered--stage 3 is of zero length). Accrual rates were set to 250 and 500 patients per unit time. The results are shown in Table 2. The estimates of *c *range between 0.63 and 0.73 (mean 0.67). Although not precisely constant, *c *does not vary greatly.

**Table 2:** Estimation of the attenuation factor, *c*, required to compute the correlations, *R_is_*, between hazard ratios on the *I*-outcome and *D*-outcome.

Acc rate	***α***_**1**_**, *α***_**2**_***, α***_**3**_			**Under *H***_**1**_				**Under *H***_**0**_			
				*R*_13_	*c*	*R*_23_	*c*	*R*_13_	*c*	*R*_23_	*c*
250	0.5, 0.25, 0.025	0.526	0.728	0.361	0.69	0.493	0.68	0.367	0.70	0.504	0.69
	0.2, 0.1, 0.025	0.776	0.907	0.529	0.68	0.594	0.66	0.529	0.68	0.598	0.66
500	0.5, 0.25, 0.025	0.527	0.728	0.369	0.70	0.476	0.64	0.383	0.73	0.487	0.67
	0.2, 0.1, 0.025	0.778	0.909	0.505	0.65	0.575	0.63	0.512	0.66	0.577	0.63

"Acc. rate" denotes the accrual rate of patients per unit time

The correlation between  and  at the end of stage 1 and at the end of stage 2 was approximately 0.6, i.e. about 10 percent smaller than *c*. As a rule of thumb, we suggest using eqn. (8) with *c *≃ 1.1  when an estimate of the correlation is available. In the absence of such knowledge, we suggest performing a sensitivity analysis of *α *and *ω *to *c *over a sensible range, for example ; see Table Seven for an example.

### 2.8 Determining 'stagewise' significance level and power

The significance level or power at stage *i *is conditional on the experimental arm *E *having passed stage *i *- 1. Let *α*_*i*|*i*-1 _be the probability under *H*_0 _of rejecting *H*_0 _at stage *i*, given that *E *has passed stage *i *- 1. Similarly, let *ω*_*i*|*i*-1 _be the 'stagewise' power, that is the probability under *H*_1 _of rejecting *H*_0 _at significance level *α_i _*at stage *i*, given that *E *has passed stage *i *- 1. Passing stage *i *- 1 implies having passed earlier stages *i*-2, *i*-3, ..., 1 as well. The motivation for calculating theoretical values of *α*_*i*|*i*-1 _and *ω*_*i*|*i*-1 _is to enable comparison with their empirical values in simulation studies.

By the rules of conditional probability, we have(9)

where *R*^(*i*) ^denotes the matrix comprising the first *i *rows and columns of *R*. *R*^(1) ^is redundant; when *i *= 2, the denominators of (9) for *α*_2|1 _and *ω*_2|1 _are *α*_1 _and *ω*_1 _respectively.

For example, suppose that *s *= 2, *α*_1 _= 0.25, *α*_2 _= 0.025, *ω*_1 _= 0.95, *ω*_2 _= 0.90,  = 0.6; then *α*_2|1 _= 0.081, *ω*_2|1 _= 0.920.

## 3 Comments on other approaches

### 3.1 Beta spending functions

Pampallona et al. [6] propose beta spending functions which allow for early stopping in favour of the null hypothesis, i.e. for lack of benefit. The beta spending functions and their corresponding critical values are derived together with alpha spending functions and hence allow stopping for benefit or futility in the same trial. An upper and a lower critical value for the hazard ratio are applied at each interim analysis. The approach is implemented in EAST5 (see http://www.cytel.com/software/east.aspx). The method may also be applied to designs which allow stopping only for lack of benefit, which is closest in spirit to our approach.

The main difference between our approach and beta spending functions lies in the specification of the critical hazard ratio, *δ_i_*, at the *i*th stage. If a treatment is as good as specified in the alternative hypothesis, we want a high probability that it will proceed to the next stage of accrual—hence the need for high power (e.g. 95%) in the intermediate stages. The only way to increase power with a given number of patients is to increase the significance level. A higher than usual significance level (*α_i_*) is justifiable because an 'error' of continuing to the next stage when the treatment arm should fail the test on *δ_i _*is less severe than stopping recruitment to an effective treatment.

Critical values for beta spending functions are determined by the shape of the spending function as information accumulates. Pampallona et al. [6]'s beta spending functions, allowing for early stopping only in favour of the null hypothesis, maintain reasonable overall power. However, a stringent significance level operates at the earlier stages, implying that the critical value for each stage is far away from a hazard ratio of 1 (the null hypothesis). Regardless of the shape of the chosen beta spending function, analyses of the intermediate outcome are conducted at a later point in time, that is, when more events have accrued, than with our approach for comparable designs.

The available range of spending functions with known properties does not allow the same power (or *α*) to be specified at two or more analyses [11]. Specifying the same power at each intermediate stage, an option in a TAMS design, is appealing because it allows the same low probability of inappropriately rejecting an effective treatment to be maintained at all stages.

### 3.2 Interim monitoring rules for lack of benefit

Recently, Freidlin et al. [12] proposed the following rule: stop for lack of benefit if at any point during the trial the approximate 95% confidence interval for the hazard ratio excludes the design hazard ratio under *H*_1_. They modify the rule (i) to start monitoring at a minimum cumulative fraction of information (i.e. the ratio of the cumulative number of events so far observed to the designed number), and (ii) to prevent the implicit hazard-ratio cut-off, *δ*, being too far below 1. (They suggest applying a similar rule to monitor for harm, that is, for the treatment effect being in the 'wrong' direction.) They state that the cost of their scheme in terms of reduced power is small, of the order of 1%.

For example, consider a trial design with Δ^1 ^= 0.75, one-sided *α *= 0.025 and power *ω *= 0.9 or 0.8. In their Tables 3 and 4, Freidlin et al. [12] report that on average their monitoring rule with 3 looks stops such trials for lack of benefit under *H*_0 _at 64% or 70% of information, respectively. The information values are claimed to be lower (i.e. better) than those from competing methods they consider. For comparison, we computed the average information fractions in simulations of TAMS designs. We studied stopping under *H*_0 _in four-stage (i.e. 3 looks) TAMS trials with *α *values of 0.5, 0.25, 0.1 and 0.025, and power 0.95 in the first 3 stages and 0.9 in the final stage. With an accrual rate of 250 pts/year, we found the mean information fractions on stopping to be 49% for designs with *I *= *D *and 21% with *I *≠ *D*. In the latter case, the hazard for *I *outcomes was twice that for *D *outcomes, resulting in greater than a halving of the information fraction at stopping compared with *I *= *D*.

As seen in the above example, a critical advantage of our design, not available with beta spending function methodology or with Freidlin's monitoring schemes, is the use of a suitable intermediate outcome measure to shorten the time needed to detect ineffective treatments. Even in the *I *= *D *case, our designs are still highly competitive and have many appealing aspects.

## 4 Simulation studies

### 4.1 Simulating realistic intermediate and definitive outcome measures

Simulations were conducted to assess the accuracy of the calculated power and significance level at each stage of a TAMS design and overall. We aimed to simulate time to disease progression (*X*) and time to death (*Y*) in an acceptably realistic way. The intermediate outcome measure of time to disease progression or death is then defined as *Z *= min (*X*, *Y*). Thus *Z *mimics the time to an *I*-event and *Y *the time to a *D*-event. Note that *X*, the time to progression, could in theory occur 'after death' (i.e. *X *>*Y*); in practice, cancer patients sometimes die before disease progression has been clinically detected, so that the outcome *Z *= min (*X*, *Y*) = *Y *in such cases is perfectly reasonable.

The theory presented by Royston et al [7] and extended here to more than 2 stages is based on the assumption that *Y *and *Z *are exponentially distributed and positively correlated. As already noted, the exponential assumption affects the values only of the stage times, *t_i_*. To generate pseudo-random variables *X*, *Y *and *Z *with the required property for *Y *and *Z*, we took the following approach. We started by simulating random variables (*U*, *V*) from a standard bivariate normal distribution with correlation *ρ*_*U*,*V *_> 0. *X *and *Y *were calculated as

where Φ is the standard normal distribution function and *λ*_1 _and *λ*_2 _are the hazards of the (correlated) exponential distributions *X *and *Y*, for which the median survival times are ln (2)/*λ*_1 _and ln (2)/*λ*_2_, respectively. Although it is well known that min (*X*, *Y*) is an exponentially distributed random variable when *X *and *Y *are independent exponentials, the same result does not hold in general for correlated exponentials.

First, it was necessary to approximate the hazard, *λ*_3_, of *Z *as a function of *λ*_1_, *λ*_2 _and *ρ*_*U*,*V*_. The approximation was done empirically by using simulation and smoothing, taking the hazard of the distribution of *Z *as the reciprocal of its sample mean. In practice, since *X *is not always observable, one would specify the hazards (or median survival times) of *Z *and *Y*, not of *X *and *Y*; the final step, therefore, was to use numerical methods to obtain *λ*_1 _given *λ*_2_, *λ*_3 _and *ρ*_*U*,*V*_.

Second, the distribution of *Z *turned out to be close to, but slightly different from exponential. A correction was applied by modelling the distribution of *W *= Φ^-1 ^[exp (-*λ*_3_*Z*)] (i.e. a variate that would be distributed as *N *(0, 1) if *Z *were exponential with hazard *λ*_3_) and finally back-transforming *W *to *Z'*, its equivalent on the exponential scale. The distribution of *W *was approximated using a three-parameter exponential-normal model [13]. Except at very low values of *Z*, we found that *Z' *<*Z*, so the correction (which was small) tended to bring the *I*-event forward a little in time.

### 4.2 Single-stage trials

A single, exponentially distributed time-to-event outcome was used in these simulations. The aim was simply to evaluate the accuracy of the basic calculation of operating characteristics outlined in sections 2.2 and 2.3. The actual type 1 error rate  and power  were estimated in the context of designs with nominal one-sided significance level *α*_1 _= {0.5, 0.25, 0.1, 0.05, 0.025} and power *ω*_1 _= {0.9, 0.95, 0.99}. Fixed single values of the allocation ratio (*A *= 1), accrual rate (*r*_1 _= 500) and hazard ratio under  and  were used. Fifty thousand replications of each combination of parameter values were generated. The Monte Carlo standard errors were  = {0.0022, 0.0019, 0.0013, 0.0010, 0.0007},  = {0.0013, 0.0010, 0.0004}. The results are shown in Table 3.The results show that the nominal significance level and power agree fairly well, but not perfectly, with the simulation results. The latter are generally larger than the former by an amount that diminishes as the sample size (total number of events) increases.

**Table 3:** Type 1 error and power for various single-stage trial designs with one-sided significance level *α*_1 _and power *ω*_1_.

Sig. Level	*ω*_1 _= 0.9		*ω*_1 _= 0.95		*ω*_1 _= 0.99	
*α*_1_						
0.5	0.516	0.918	0.506	0.960	0.503	0.993
0.25	0.256	0.908	0.257	0.956	0.250	0.992
0.1	0.105	0.906	0.104	0.955	0.104	0.992
0.05	0.054	0.906	0.054	0.954	0.053	0.991
0.025	0.029	0.903	0.028	0.954	0.027	0.991

The hazard ratio under *H*_1 _was fixed at 0.75

The causes of the inaccuracies in *α*_1 _and *ω*_1 _are explored in Appendix C. The principal reason for the discrepancy in the type 1 error rate  is that the estimate of the variance of the log hazard ratio under *H*_0 _given in equation (3) is biased downwards by up to about 1 to 3 percent. Regarding the power, the estimate of the variance of the log hazard ratio under *H*_1 _given in equation (5) is biased upwards by up to about 4 percent. For practical purposes, however, we consider that the accuracy levels are acceptable, and we have not attempted to further correct the estimated variances.

### 4.3 Multi-stage trials

#### 4.3.1 Design

We consider only designs for TAMS trials with 3 stages. We report the actual stagewise and overall significance level and power, comparing them with theoretical values derived from multivariate normal distribution as given in eqns. (6) and (9). Actual significance levels were estimated from simulations run under *H*_0 _with hazard ratio  = 1 (*i *= 1, ..., *s*). Power was estimated from simulations run under *H*_1 _with hazard ratio  = 0.75 (*i *= 1, ..., *s*). Other design parameter values were based on those used in the GOG182/ICON5 two-stage trial, taking median survival for the *I*-outcome, progression-free survival, of 1 yr (hazard *λ*_1 _= 0.693), and for the *D*-outcome, survival, of 2 yr (hazard *λ*_2 _= 0.347). Correlations among hazard ratios at the intermediate stages, *R_ij_*, were computed from eqn. (7) for *i*, *j *<*s*. Values of *R_is _*(*i *= 1, ..., *s*-1) were estimated as the empirical correlations between  and  in an independent set of simulations of the relevant design scenarios. Three designs were used: *α_i _*= {0.5, 0.25, 0.025}, {0.2, 0.1, 0.025}, {0.1, 0.05, 0.025} with *ω_i _*= {0.95, 0.95, 0.9} in each case.

Simulations were performed in Stata using 50,000 replications of each design. Pseudo-random times to event *X*, *Y *and *Z' *were generated as described in section 4.1.

#### 4.3.2 Results

Tables 4(a) and 4(b) give simulation results for 3 three-stage trial designs with accrual rates of 250 and 500 patients per year, respectively.

**Table 4:** Simulation results (50,000 replicates) for 3 three-stage trial designs with accrual rates (*r*_*i*_) of (a) 250 and (b) 500 patients per year.

Design	Stage	*α_i_*	*ω_i_*	*δ_i_*	*e_i_*	*t_i_*	*N_i_*	*α*_*i*|*i*-1_		*ω*_*i*|*i*-1_	
(a) *r_i _*= 250											
1	1	0.50	0.95	1.000	73	1.53	191	0.500	0.495	0.950	0.957
	2	0.25	0.95	0.923	140	0.74	283	0.441	0.452	0.969	0.971
	3	0.025	0.90	0.843	264	2.10	545	0.074	0.084	0.918	0.923

2	1	0.2	0.95	0.910	159	2.45	306	0.200	0.204	0.950	0.955
	2	0.1	0.95	0.885	217	0.55	375	0.427	0.432	0.976	0.978
	3	0.025	0.90	0.844	264	1.36	545	0.144	0.158	0.924	0.930

3	1	0.1	0.95	0.885	217	3.00	375	0.100	0.104	0.950	0.953
	2	0.05	0.95	0.869	272	0.49	436	0.423	0.431	0.980	0.981
	3	0.025	0.90	0.844	264	0.87	545	0.221	0.243	0.926	0.932

(b) *r_i _*= 500											
1	1	0.50	0.95	1.000	74	1.03	259	0.500	0.503	0.950	0.957
	2	0.25	0.95	0.923	141	0.46	374	0.441	0.447	0.969	0.971
	3	0.025	0.90	0.844	266	1.40	722	0.074	0.084	0.918	0.925

2	1	0.2	0.95	0.910	161	1.62	404	0.200	0.203	0.950	0.954
	2	0.1	0.95	0.885	220	0.33	487	0.427	0.439	0.976	0.979
	3	0.025	0.90	0.844	266	0.94	722	0.144	0.150	0.924	0.927

3	1	0.1	0.95	0.885	220	1.95	487	0.100	0.103	0.950	0.954
	2	0.05	0.95	0.869	275	0.29	559	0.423	0.433	0.980	0.982
	3	0.025	0.90	0.844	266	0.65	722	0.221	0.224	0.926	0.929

Median survival times are 1 year for the *I*-outcome and 2 years for the *D*-outcome. Hazard ratio is 1.0 under *H*_0 _and 0.75 under *H*_1_.

Key: *i*, stage; *α_i_*, nominal significance level at stage *i*; *ω_i_*, nominal power at stage *i*; *δ_i_*, cut-off for HR--experimental arm passes to stage *i *+ 1 (or, if *i *= *s*, is declared significant) if  < δ*_i_*; *r_i_*, rate of patient accrual per year during stage *i*; *e_i_*, cumulative number of control arm events required at end of stage *i*; *t_i_*, duration (in years) of stage *i*; *N_i_*, cumulative number of patients accrued to control arm by end of stage *i*; *α*_*i*|*i*-1_, 'stagewise' significance level, i.e. significance level at stage *i *given that experimental arm has passed stage *i *- 1; *ω*_*i*|*i*-1_, 'stagewise' power, i.e. power at stage *i *given that experimental arm has passed stage *i *- 1.

Only the columns labelled  and  are estimates from simulation. The remaining quantities are either primary design parameters (*r_i_*, *α_i_*, *ω_i_*) or secondary design parameters (*δ_i_*, *e_i_*, *t_i_*, *N_i_*). The latter are derived from the former according to the methods described in section 2, additionally with . Note that by convention *α*_1|0 _= *α*_1 _and *ω*_1|0 _= *ω*_1_, the corresponding estimates  being, respectively, the empirical significance level and power at stage 1. Monte Carlo standard errors for underlying probabilities of {0.95, 0.90, 0.5, 0.25, 0.10, 0.05} with 50,000 replications are approximately {0.00097, 0.0013, 0.0022, 0.0019, 0.0013, 0.00097}. The results show good agreement between nominal and simulation values of  and , but again with a small and unimportant tendency for the simulation values to exceed the nominal ones.

Table 5 presents the overall significance level and power for the designs in Table 4, with (*α*, *ω*) as predicted from a trivariate normal distribution and  as estimated by simulation.

**Table 5:** Overall significance level and power for the three-stage trial designs presented in Table 4.

Accrual	Design	*α*		*ω*	
*r_i _*= 250	1	0.016	0.019	0.845	0.858
	2	0.012	0.014	0.857	0.869
	3	0.009	0.011	0.862	0.871

*r_i _*= 500	1	0.016	0.019	0.845	0.861
	2	0.012	0.013	0.857	0.866
	3	0.009	0.010	0.862	0.871

See text for further details.

The same tendencies are seen as in the earlier tables. The calculated values of the overall significance level and power both slightly underestimate the actual values.

## 5 Example in prostate cancer: the STAMPEDE trial

STAMPEDE is a MAMS trial conducted at the MRC Clinical Trials Unit in men with prostate cancer. The aim is to assess 3 alternative classes of treatments in men starting androgen suppression. In a four-stage design, five experimental arms with compounds shown to be safe to administer are compared with a control arm regimen of androgen suppression alone. Stages 1 to 3 utilize an *I*-outcome of failure-free survival (FFS). The primary analysis is carried out at stage 4, with overall survival (OS) as the *D*-outcome.

As we have already stated, the main difference between a MAMS and a TAMS design is that the former has multiple experimental arms, each compared pairwise with control, whereas the latter has only one experimental arm. The design parameters for MAMS and TAMS trials are therefore the same.

For STAMPEDE, the design parameters, operating characteristics, number of control-arm events and time of the end of each stage are shown in Table 6.

**Table 6:** STAMPEDE design parameters.

Stage (*i*)	Outcome	*α_i_*	*ω_i_*	*δ_i_*	*e_i_*	*t_i_*
1	FFS	0.5	0.95	1.00	113	3.0
2	FFS	0.25	0.95	0.92	213	4.4
3	FFS	0.1	0.95	0.89	331	5.8
4	OS	0.025	0.9	0.84	403	8.0

Overall		0.017	0.84*			
		0.012	0.83**			

*Using corr. matrix *R*_1_

**Using corr. matrix *R*_2_

Time is expressed in years. Accrual rate (*r_i_*) was planned to be 348 patients per year in each stage. FFS = failure-free survival, OS = overall survival.

Originally, a correlation matrix *R*_1_, defined by eqn. (6) and taking the *e_i _*from Table 6, was used to calculate the overall significance level and power:

*R*_1 _was an 'educated guess' at the correlation structure. An alternative, *R*_2_, which uses eqns. (7) and (8) with *c *= 0.67 (also an educated guess), is

The overall significance level and power are slightly lower with *R*_2 _than with *R*_1 _(Table 6). To explore the effect of varying *c *and *R*, in Table 7 we present a sensitivity analysis of the values of *α *and *ω *to the choice of *c*. [The values of *α *and *ω *in Table 7 were calculated using eqns (7) and (8). The significance level varies by a factor of about 2 over the chosen range of *c*, whereas the power is largely insensitive to *c*. We believe that [0.4, 0.8] is a plausible range for *c *in general. Note that (*α*, *ω*) are bounded above by (*α_s_*, *ω_s_*)--here, by (0.025, 0.9). Thus the overall one-sided significance level for a treatment comparison is guaranteed to be no larger than 0.025 and is likely to be considerably smaller. The overall power is likely to lie in the range [0.82, 0.84] and cannot exceed 0.9.

**Table 7:** Sensitivity of the overall significance level (*α*) and power (*ω*) of pairwise comparisons with the control arm in the STAMPEDE design to the choice of the constant *c*.

*c*	*α*	*ω*
0.4	0.0067	0.822
0.5	0.0084	0.826
0.6	0.0104	0.830
0.7	0.0127	0.835
0.8	0.0153	0.841

As a general rule, the values in Table 7 suggest that it may be better to underestimate rather than overestimate *c *as this would lead to conservative estimates of the overall power.

As illustrated in Table 6, larger significance levels *α_i _*were chosen for stages 1-3 than would routinely be considered in a traditional trial design. The aim was to avoid rejecting a potentially promising treatment arm too early in the trial, while at the same time maintaining a reasonable chance of rejecting treatments with hazard ratio worse than (i.e. higher than) the critical value *δ_i_*.

## 6 Discussion

The methodology presented in this paper aims to address the pressing need for new additions to the 'product development toolkit' [1] for clinical trials to achieve reliable results more quickly. The approach compares a new treatment against a control treatment on an intermediate outcome measure at several stages, allowing early stopping for lack of benefit. The intermediate outcome measure does not need to be a surrogate for the primary outcome measure in the sense of Prentice [14]. It does need to be related in the sense that if a new treatment has little or no effect on the intermediate outcome measure then it will probably have little or no effect on the primary outcome measure. However, the relationship does not need to work in the other direction; it is not stipulated that because an effect has been observed on the intermediate outcome measure, an effect will also be seen on the primary outcome measure. A good example of an intermediate outcome is progression-free survival in cancer, when overall survival is the definitive outcome. Such a design, in two stages only, was proposed by Royston et al. [7] in the setting of a multi-arm trial. In the present paper, we have extended the design to more than two stages, developing and generalizing the mathematics as necessary.

In the sample size calculations presented here, times to event are assumed to be exponentially distributed. Such an assumption is not realistic in general. In the TAMS design, an incorrect assumption of exponential time-to-event affects the timelines of the stages, but under proportional hazards of the treatment effect, it has no effect on the numbers of events required at each stage. A possible option for extending the method to non-exponential survival is to assume piecewise exponential distributions. The implementation of this methodology for the case of parallel group trials was described by Barthel et al. [15]. Further work is required to incorporate it into the multi-stage framework.

Another option is to allow the user to supply the baseline (control arm) survival distribution seen in previous trial(s). By transforming the time-to-event into an estimate of the baseline cumulative hazard function, which has a unit exponential distribution, essentially the same sample size calculations can be made, regardless of the form of the actual distribution. 'Real' timelines for the stages of the trial can be obtained by back-transformation, using flexible parametric survival modelling [16] implemented in Stata routines [17,18] The only problem is that the patient accrual rate, assumed constant (per stage) on the original time scale, is not constant on the transformed time scale; it is a continuous function of the latter. The expression for the expected event rate *e *(*t*) given in eqn. (10) is therefore no longer valid, and further extension of the mathematics in Appendix A is needed. This is another topic for further research.

We used simulation to assess the operating characteristics of TAMS trials based on a bivariate exponential distribution, obtained by transforming a standard bivariate normal distribution. The simulation results confirm the design calculations in terms of the significance level and power actually attained. They show that overall power is maintained at an acceptable level when adding further stages.

Multi-stage trials and the use of intermediate outcomes are not new ideas. Trials with several interim analyses and stopping rules have been suggested in the context of alpha and beta spending functions. Posch et al. [19] have reviewed the ideas. One of the main differences between other approaches and ours is the method of calculation of the critical value for the hazard ratio at each stage or interim analysis, as discussed in section 3. With the error spending-function approach, the critical value is driven by the shape chosen for the function. In our approach, it is based on being unable to reject *H*_0 _at modest significance levels.

Our approach differs from that of calculating conditional power for futility. In the latter type of interim analysis, the conditional probability of whether a particular clinical trial is likely to yield a significant result in the future is assessed, given the data available so far [2]. *Z*-score boundaries are plotted based on conditional power and on the information fraction at each point in time. These values must be exceeded for the trial to stop early for futility. In contrast, we base the critical value at each stage not on what may happen in the future, but rather on the data gathered so far.

We note that further theoretical development of TAMS designs is required. Questions to be addressed include the following. (1) How do we specify the stagewise significance levels (*α_i_*) and power (*ω_i_*) to achieve efficient designs (e.g. in terms of minimizing the expected number of patients)? We have made some tentative suggestions in section 2.6, but a more systematic approach is desirable. (2) Given the uncertainty of the correlation structure of the treatment effects on the different types of outcome measure (see section 2.7.1), what are the implications for the overall significance level and power?

In the meantime, multi-arm versions of TAMS trials have been implemented in the real world, and new ones are being planned. We believe that they offer a valuable way forward in the struggle efficiently to identify and evaluate the many potentially exciting new treatments now becoming available. Further theoretical developments will follow as practical issues arise.

## 7 Conclusions

We describe a new class of multi-stage trial designs incorporating repeated tests for lack of additional efficacy of a new treatment compared with a control regimen. Importantly, the stages include testing for lack of benefit with respect to an intermediate outcome measure at a relaxed significance level. If carefully selected, such an intermediate outcome measure can provide more power and consequently a markedly increased lead time. We demonstrate the mathematical calculation of the operating characteristics of the designs, and verify the calculations through computer simulations. We believe these designs represent a significant step forward in the potential for speeding up the evaluation of new treatment regimens in phase III trials.

## 8 Appendix A. Further details of algorithms for sample size Calculations

As noted in section 2.4, two subsidiary algorithms are needed in the sample size calculations for a TAMS trial. We adopt the following notation and assumptions:

• Calendar time is denoted by *t*. The start of the trial (i.e. beginning of recruitment) occurs at *t *= 0.

• No patient drops out or is lost to follow-up

• Stages 1, ..., *s *start at *t*_0_, ..., *t*_*s*-1 _and end at *t*_1_, ..., *t_s _*time-units (e.g. years), respectively. We assume that *t*_0 _= 0 and *t*_*i*-1 _<*t_i _*(*i *= 1, ..., *s*).

• Duration of stage *i *is *d_i _*= *t_i _*- *t*_*i*-1 _time-units.

• Recruitment occurs at a uniform rate in each stage, but the rate may vary between stages. The number of patients recruited to the control arm during stage *i *is *r_i_*.

• Number of events expected in interval (0, *t*] = *e*(*t*).

• Survival function is *S *(*t*) and distribution function is *F *(*t*) = 1 - *S *(*t*)

• Number of patients at risk of an event at time *t *= *N*(*t*), with *N *(0) = 0

If patients are recruited at a uniform rate, *r *per unit time, in an interval (0, *t*], the expected number of events in that interval is(10)

### 8.1 Determining the numbers of events from the stage times

Step 4 of the sample size algorithm requires calculation of the number of events expected at the end of a stage, given the recruitment history up to that point. Consider *N *(*t*_1_), the number of patients at risk of an event at the end of stage 1. Assuming no drop-out, this is given by (number of patients recruited in stage 1) minus (expected number of events in (0, *t*_1_]), that is

To compute *N *(*t*_2_), we consider two subsets of patients: the *N *(*t*_1_) patients recruited during stage 1 and still at risk at *t*_1_, and the *r*_2 _(*t*_2 _- *t*_1_) new patients recruited during stage 2, i.e. in (*t*_1_, *t*_2_]. Provided the survival distribution is 'memoryless' (e.g. the exponential distribution), the number of 'survivors' from the first subset at *t*_2 _is *N *(*t*_1_) *S *(*t*_2 _- *t*_1_). In this case we have

Generalizing this expression for stage *i *(*i *= 1, ..., *s*) as a recurrence relation convenient for computer evaluation, we have(11)

Regarding *e *(*t*), the expected number of events, we can derive, by a similar argument, the recurrence relation(12)

for *i *= 1, ..., *s*. Equations (11) and (12) enable the calculation of the number of patients at risk and number of events at the end of any stage for a memoryless survival distribution under the assumption of a constant recruitment rate in each stage.

If the survival distribution is exponential with hazard *λ*, the required functions of *t *are

In general terms, the numbers at risk and expected numbers of events at any given stage may be computed using (11) and (12). Write *e *(*t_i_*) = *e *(*t_i_*; *λ*) to emphasize the dependence on the hazard in the case of the exponential distribution. Let *λ_I _*and *λ_D _*be the hazards for *I*-events and *D*-events, respectively. In the notation of section 2.4, we have

### 8.2 Calculating times from cumulative events

Step 3 of section 2.4 involves computing the stage endpoints given the number of events occurring in each stage. This may be done by using a straightforward Newton-Raphson iterative scheme.

Consider a function *g *(*x*). We wish to find a root *x *such that *g *(*x*) ≈ 0. The Newton-Raphson scheme requires a starting guess, *x*^(0)^. The next guess is given by *x*^(1) ^= *x*^(0) ^- *g *(*x*^(0)^)/*g' *(*x*^(0)^). The process continues until some *i *is found such that |*x*^(*i*) ^- *x*^(*i*-1)^| is sufficiently small. In well-behaved problems, convergence is fast (quadratic) and unique.

Given a cumulative number of events, *e*, we wish to find *t *such that *e *(*t*) ≈ *e*, i.e. *t *such that *g *(*t*) = *e*-*e *(*t*) ≈ 0. Suppose we have a vector (*e*_1_, ..., *e_s_*) of events whose corresponding times (*t*_1_, ..., *t_s_*) are to be found, and that the first *i *- 1 times have been found to be *t*_1_, ..., *t*_*i*-1_. To find *t_i_*, we have

with *N *(*t*_*i*-1_) given by (11) and *e *(*t_i_*) by eqn. (12). Hence

For the exponential distribution, we have

A reasonable starting value for *t_i _*is *t*_*i*-1 _+ 0.5× median survival time. Updates of *t_i _*are performed in routine fashion using the Newton-Raphson scheme. Adequate convergence usually occurs within about 8 iterations.

### 8.3 Stopping recruitment before the end of stage *s*

We turn to the situation where recruitment is stopped at some time t* <*t_s_*, and all recruited patients are followed up for events until *t_s_*. This may be a good option when recruitment is slow, at the cost of increasing the length of the trial. Let *a *∈ {0, 1, ..., *s *- 1} be the stage immediately preceding the time t*, that is, t* occurs during stage *t*_*a*+1 _so that t* ∈ (*t_a_*, *t*_*a*+1_]. If *a *= 0, for example, recruitment ceases before the end of stage 1. We assume that the recruitment rate is *r*_*a*+1 _between *t_a _*and t* and zero between t* and *t*_*a*+1_. Let d* = t* - *t_a _*be the duration of recruitment during stage *a *+ 1. In practice, as explained in section 2.5, we restrict the application of these formulae to the case *a *+ 1 = *s*.

We now consider the extension of the calculations to allow early stopping of recruitment for the cases in steps 4 and 3 of the sample size algorithm described in section 2.4.

#### 8.3.1 Step 4: Determining the number of events from the stage times

By arguments similar to those in section 8.1, we have(13)(14)

In fact, *e *(t*) is the expected number of events at an arbitrary timepoint t* ∈ (0, *t_s_*). The total number of patients recruited to the trial is .

#### 8.3.2 Step 3: Calculating times from cumulative events

Given *a *and t*, numbers of events *e*_1_, ..., *e_a_*, *e*_*a*+1 _and stage endpoints *t*_1_, ..., *t_a_*, we wish to find *t*_*a*+1 _to give *e*_*a*+1 _cumulative events. Similar to section 8.1, we have

where *N *(t*) and *e *(t*) are as given in eqns. (13) and (14).

For determining the unknown *t*_*a*+1 _by Newton-Raphson iteration, the only term in *e*_*a*+1 _that includes the 'target' value *t*_*a*+1 _is *N *(t*) *F *(*t*_*a*+1 _- t*). For the exponential distribution, the derivative of *N *(t*) *F *(*t*_*a*+1 _- t*) with respect to *t *at *t*_*a*+1 _is *N *(t*) *λ *[1 - *F *(*t*_*a*+1 _- t*)], so that

The iterative scheme may be applied as in section 8.2 to solve for *t*_*a*+1_.

## 9 Appendix B. Determining the correlation matrix (*R_ij_*)

### 9.1 Approximate results

We assume that the arrivals of patients into the trial follow independent homogeneous Poisson processes with rates *r *in the control arm and *Ar *in the experimental arm, where *A *is the allocation ratio. This is equivalent to patients entering the trial in a Poisson process of rate (1 + *A*)*r *and being assigned independently to *E *(the experimental arm) with probability *p *= *A*/(1 + *A*) or to *C *(the control arm) with probability 1 - *p *= 1/(1 + *A*).

If, for each arm, the intervals between entry of the patient into the trial and the event of interest (analysis times) are independent and identically distributed, and if we ignore the effect of initial conditions (the start of the trial at *t *= 0) so that the process of events occurring in each arm is in equilibrium, these events occur in Poisson processes with rates *r *and *Ar *in the two arms. If, additionally the two sequences of intervals are independent, then the two Poisson processes are also independent. Note that there is no requirement here that the analysis times (i.e. the intervals between patient entries and event-times) have the same distribution for patients in both arms of the trial.

In the following discussion in this section, we consider the equilibrium case under the above assumptions. The transient case is deferred to section 9.2.

We begin observing events in each arm at *t *= 0. We await *m*_1 _events in the control arm at time *T*_1 _(stage 1), a *further m*_2 _events during the subsequent time period of length *T*_2 _(stage 2), and so on up to stage *s*. Thus we await *e_i _*= *m*_1 _+*m*_2 _+ ... +*m_i _*control-arm events by time *t_i _*= *T*_1 _+*T*_2 _+ ... +*T_i _*(stage *i*). Quantities *m_i _*(*i *= 1, ..., *s*) are fixed whereas {*T_i_*, *i *= 1, ..., *s*} are mutually independent random variables, where *T_i _*has a gamma distribution, Γ (*m_i_*, *r*), with index *m_i _*and scale parameter *r*.

Let the number of events observed in the experimental arm at *T*_1 _be *O*_1 _and the incremental numbers of events observed in the experimental arm during the subsequent time periods of lengths *T*_2_, ..., *T_s _*be *O*_2_, ...,*O_s _*respectively. Given {*T_i_*, *i *= 1, ..., *s*}, the variables {*O_i_*} are mutually independent, where *O_i _*has a Poisson distribution with rate *Ar *and mean *ArT_i_*. Since the {*T_i_*} are mutually independent, the same is true of the {*O_i_*} unconditionally.

Let the random variable *N_c _*(*t*) be the number of control-arm events observed by time *t*. The parameter Δ*_i _*denotes the hazard ratio at stage *i*. Then, at stage 1, the hazard ratio is

More generally, for *i *= 1, ..., *s*, at stage *i *the hazard ratio is

For 1 ≤ *i *<*j *≤ *s *we require the correlation

as correlations are invariant under linear transformations of the variables.

Since the *O_i _*are mutually independent, it follows that

We determine this correlation for the case *i *= 1, *j *= 2; the derivation for general *i *and *j *is the same. It is easy to see that

and similarly that

It follows that

and more generally that for 1 ≤ *i *≤ *j *≤ *s*(15)

Equation (15) gives the correlation between the hazard ratios when it is assumed that the processes of events in the two arms are in equilibrium. In the next section, we show that the equilibrium result given in equation (15) holds exactly in the non-equilibrium case when the distributions of the intervals between trial entry and event are the same for the two arms of the trial. In this case, the result is easily derived under the more general assumption that the Poisson process of trial entries is nonstationary. In section 9.3, a comparison is made with exact correlations estimated by simulation for a typical example.

### 9.2 Exact results

We now suppose that the trial begins at *t *= 0, with no entries into either arm before that time. For simplicity of notation, we will focus on *s *= 2; the extension to larger values of *s *is straightforward. We assume that entries into the trial form a Poisson process with rate (1 + *A*)*r*(*t*)(*t *> 0) and, as before, are independently allocated to the experimental and control arms with probabilities *p *= *A*/(1 + *A*) and 1 - *p *respectively.

In the experimental arm, if analysis times are independent and identically distributed with common density *f_e_*, the events form another (nonhomogeneous) Poisson process with rate

again starting from *t *= 0. Thus, *O*_1 _has a Poisson distribution with mean *Aθ_e_*(*T*_1_), where

Similarly, *O*_1 _and *O*_2 _are independent Poisson variables and *O*_1 _+ *O*_2 _has a Poisson distribution with mean *Aθ_e_*(*T*_1 _+ *T*_2_).

For the control arm, if the analysis times have density *f_c _*and we define

then the mean numbers of events in (0, *T*_1_] and (0, *T*_1 _+ *T*_2_] are *θ_c_*(*T*_1_) and *θ_c_*(*T*_1 _+ *T*_2_).

Thus the hazard ratio parameters are

Under the hypothesis that the densities *f_e _*and *f_c _*are the same in the two arms of the trial (as is typically the case under the null hypothesis, Δ = 1), the two functions *θ_e _*and *θ_c _*coincide and the hazard ratios simplify. It is then straightforward to see that, as in the equilibrium analysis,

where var(*O*) = *E*(*Aθ_e_*(*T*))+var(*Aθ_e_*(*T*)), and *O *denotes the observed number of events in the experimental arm in an arbitrary time *T*.

Suppose that *T *is the time elapsing until the *m*th event in the control arm. Then, *T *>*t *if and only if *N_c_*(*t*) <*m*. As *N_c_*(*t*) has a Poisson distribution with mean *θ_c_*(*t*),

from which it follows that *T *has density

and therefore that the random variable *θ_c_*(*T*) has a gamma distribution Γ(*m*, 1) with index *m *and scale parameter 1. Note that, by transforming the time scale from *t *to *θ_c_*(*t*) we are transforming to *operational time *(see Cox and Isham [20], section 4.2), in which events in the control arm occur in a Poisson process of unit rate. The method works here because the transformed time scales are, up to the constant *A*, assumed to be the same in the two arms of the trial.

Finally, since we have assumed the equivalence of *θ_e _*and *θ_c_*, var(*O*) = *AE*(*θ_c_*(*T*)) + *A*^2^var(*θ_c_*(*T*)) = *A*(1 + *A*)*m*, and thus, as before,

### 9.3 Example

The example is loosely based on the design of the MRC STAMPEDE trial [9] in prostate cancer. We consider *s *= 4 stages and a single event-type (i.e. no intermediate event-type). We wish to compare {*R_ij_*} for *i*, *j *= 1, ..., *s *from simulation with the values derived from equation (15). At the *i*th stage, whose timing is determined by the predefined significance level *α_i _*and power *ω_i_*, the hazard ratio between the experimental and control arms is calculated and compared with a cut-off value, *δ_i_*, calculated as described in section 2.3. In practice, the number of events *e_i _*required in the control arm at the *i*th stage is computed and the analysis is performed when that number has been observed. The (one-sided) significance levels, *α_i_*, at the four stages were chosen to be 0.5, 0.25, 0.1, 0.025 and the power values, *ω_i_*, to be 0.95, 0.95, 0.95, 0.9. The allocation ratio was taken as *A *= 1. The accrual rate was assumed to be 1000 patients per year, with a median time to event (analysis time) of 4 years.

The design (see Table 8) was simulated 5000 times and the empirical Pearson correlations between the estimates  (*i *= 1, ..., 4) of the hazard ratios were computed when the underlying hazard ratio, Δ, was 1 (null hypothesis) or 0.75 (typical alternative hypothesis). The results for Δ = 1 are shown in Table 9.When Δ = 1, the exact results of section 9.2 apply, and any discrepancies in Table 9 should be due to sampling variation. The simulated values are in fact within one Monte Carlo standard error (0.014) of the theoretical values, which supports equation (15). The root mean square discrepancy across the 6 correlations is 0.0067.

**Table 8:** Parameters of the four-stage trial design used in the simulation study. See text for details

Stage(*i*)	*α_i_*	*ω_i_*	*δ_i_*	*e_i_*
1	0.5	0.95	1.000	73
2	0.25	0.95	0.923	140
3	0.1	0.95	0.884	217
4	0.025	0.9	0.843	262

**Table 9:** Estimates of correlations *R*_*ij*_. Lower triangle (in italics), based on equation (15); upper triangle, estimates based on simulation under Δ = 1, 5000 replications

*R_ij_*	*i *= 1	*i *= 2	*i *= 3	*i *= 4
*j *= 1	1	0.721	0.575	0.519
*j *= 2	*0.722*	1	0.799	0.722
*j *= 3	*0.579*	*0.802*	1	0.909
*j *= 4	*0.529*	*0.733*	*0.914*	1

When Δ = 0.75, however, we must rely on the equilibrium approximation. Any errors are a mixture of sampling variation and bias due to the use of the approximation. Simulation results are given in Table 10.The discrepancies are slightly larger than in Table 9. The root mean square discrepancy across the 6 correlations is 0.0121, about double that for Δ = 1. Nevertheless, for practical use, equation (7) provides an excellent approximation in the present scenario.

**Table 10:** Estimates of correlations *R*_*ij*_.

*R_ij_*	*i *= 1	*i *= 2	*i *= 3	*i *= 4
*j *= 1	1	0.715	0.569	0.512
*j *= 2	*0.722*	1	0.793	0.717
*j *= 3	*0.579*	*0.802*	1	0.904
*j *= 4	*0.529*	*0.733*	*0.914*	1

Lower triangle (in italics), based on equation (15); upper triangle, estimates based on simulation under Δ = 0.75, 5000 replications

Further simulations were performed with Δ = 0.50 and Δ = 0.35. The results (not shown) confirmed that equation (15) provides an excellent approximation.

## 10 Appendix C. How do the inaccuracies in power and significance level arise?

Since at stage *i*

it follows that under *H*_0_, the sampling distribution of the random variable

should have mean , variance 1, skewness 0 and kurtosis 3. Similarly, under *H*_1_,

should have mean , variance 1, skewness 0 and kurtosis 3. If the estimate  is biased, the means of *A_i _*and *B_i _*in simulation studies will differ from  and  under *H*_0 _and *H*_1_, respectively. If there is bias in the estimates of  and , the SDs of simulated values of *A_i _*and *B_i _*will differ from  and  under *H*_0 _and *H*_1_, respectively. The direction of the bias of the SD will be the opposite to that in the estimators of  and .

Table 11 shows the means and SDs of the *A_i _*for stage 1 (*i *= 1). Except for *α*_1 _= 0.5, *ω*_1 _= 0.90, the case with the smallest number of events, the bias in the mean is small and positive. The bias in the SD is larger and positive (about 1 to 3 percent), suggesting that the estimator of  in eqn. (3) is biased downwards somewhat.

**Table 11:** Means and SDs of random variable *A*_1 _for the simulations in Table 3, computed under *H*_0_

Sig. Level *α*_1_		ω_1 _= 0.90		ω_1 _= 0.95		ω_1 _= 0.99	
		Mean	SD	Mean	SD	Mean	SD
0.5	0.000	0.043	0.995	0.018	1.006	0.004	1.000
0.25	-0.674	-0.670	1.017	-0.667	1.015	-0.673	1.005
0.1	-1.282	-1.278	1.021	-1.286	1.013	-1.277	1.014
0.05	-1.645	-1.647	1.018	-1.648	1.019	-1.646	1.014
0.025	-1.960	-1.955	1.031	-1.955	1.019	-1.963	1.018

Table 12 shows the means and SDs of the *B_i _*for *i *= 1. The values of  corresponding to *ω*_1 _= 0.90, 0.95 and 0.99 are 1.282, 1.645 and 2.326, respectively. Except for *α*_1 _= 0.5, *ω*_1 _= 0.90, the bias in the mean is small and negative--about half a percent. The bias in the SD is larger and negative (about 4 percent), suggesting that the estimator (5) of  is biased upwards somewhat. 2

**Table 12:** Means and SDs of random variable *B*_1 _for the simulations in Table 3, computed under *H*_1_

Sig. Level *α*_1_		ω_1 _= 0.90		ω_1 _= 0.95		ω_1 _= 0.99	
		Mean	SD	Mean	SD	Mean	SD
0.5	0.000	1.302	0.920	1.646	0.936	2.314	0.939
0.25	-0.674	1.274	0.954	1.638	0.952	2.316	0.952
0.1	-1.282	1.273	0.963	1.630	0.962	2.316	0.963
0.05	-1.645	1.272	0.966	1.630	0.965	2.319	0.966
0.025	-1.960	1.272	0.977	1.636	0.970	2.311	0.968

## Competing interests

The authors declare that they have no competing interests.

## Authors' contributions

PR and MKBP conceived the new designs. PR, FMB and MKBP drafted the manuscript. PR, FMB and VI carried out the mathematical calculations. BCO and FMB designed and carried out the computer simulations, and tabulated the results. All authors read and approved the final manuscript.
